# Does Preoperative Virtual Reality Experience Enhance Implant Positioning Accuracy in Total Hip Arthroplasty?

**DOI:** 10.7759/cureus.70390

**Published:** 2024-09-28

**Authors:** Masaya Ueno, Shunsuke Kawano, Masanori Fujii, Shiori Tanaka, Kii Sakumo, Tadatsugu Morimoto

**Affiliations:** 1 Orthopaedic Surgery, Saga University, Saga, JPN; 2 Research Center of Arthroplasty, Saga University, Saga, JPN

**Keywords:** orthopedic surgery training, preoperative planning, surgical precision, total hip arthroplasty, virtual reality

## Abstract

Purpose

Total hip arthroplasty (THA) requires precise implant positioning to ensure long-term success. Herein, we evaluated the effect of virtual reality (VR) on the surgical precision of THA, particularly when used by experienced surgeons.

Methods

In this single-center, prospective, case-control study, 34 patients who underwent primary THA performed by a single experienced surgeon were divided into the control (without VR simulation) and VR (with VR simulation) groups. Preoperative planning involved the creation of three-dimensional models from computed tomography scans using ZedHip® software (Lexi, Tokyo, Japan). The primary outcomes assessed included the accuracy of implant placement, operative time, and intraoperative blood loss. The secondary outcomes included postoperative hospital stay and in-hospital complications.

Results

A significant improvement in radiographic inclination (RI) was observed in the VR group as compared to controls. Other surgical parameters, such as radiographic anteversion, operation time, blood loss, and postoperative hospital stay, showed no significant differences between the groups. Discrepancies in planned versus actual implant sizes were noted but were not significantly different between groups.

Conclusion

VR application in preoperative planning improved the RI accuracy in acetabular cup placement for THA, demonstrating its potential to enhance surgical precision for experienced surgeons. This study highlights the evolving role of VR, from a training tool to an integral part of advanced surgical planning in orthopedics.

## Introduction

Total hip arthroplasty (THA) is one of the most successful procedures within the field of orthopedic surgery [1,2]; however, long-term success is heavily influenced by the precise positioning of implants. The complexity of achieving optimal placement of the acetabular and femoral components underlines the need for a steep learning curve in THA [3]. Malpositioning or malalignment of the acetabular or femoral component can lead to increased dislocation, impingement, osteolysis, migration, and wear [4,5].

To enhance the precision of THA, three-dimensional (3D) preoperative planning with computed tomography (CT) has become more prominent than traditional two-dimensional (2D) X-ray-based templating. This method allows comprehensive visualization of the patient's anatomy, facilitating accurate sizing and orientation of the implants [6,7]. Furthermore, 3D planning enhances the utility of computer-assisted surgery (CAS) technologies, including navigation systems and robot-assisted procedures, which have been introduced to minimize discrepancies in the alignment of acetabular components. In recent years, many studies have demonstrated that these new technologies can significantly improve the accuracy of acetabular component placement, and contribute to more consistent restoration of leg length and offset, thus improving surgical outcomes [8-11].

In addition, recent advances in virtual reality (VR) have been integrated into surgical training using affordable head-mounted displays (HMDs). While this technology primarily benefits less experienced surgeons by offering immersive training experiences [12-16], a comprehensive literature review revealed a paucity of research evaluating the impact of VR technology on expert surgeons’ performance. Our team has approached this gap by developing a novel system that generates patient-specific 3D bone models for each THA case in a VR environment. This unique approach facilitates the precise placement of the acetabular component in individualized 3D models and explores the potential improvement of surgical precision in experienced surgeons.

We hypothesized that advanced preoperative VR technology would have a significant impact on the surgical precision of highly skilled THA surgeons, with a particular focus on enhancing their performance. Our aim was to determine whether this novel VR preparation could improve surgical outcomes, specifically the accuracy of acetabular implant placement.

## Materials and methods

This single-center, one-surgeon, prospective, case-control study was conducted in accordance with the ethical guidelines outlined in the 1975 Declaration of Helsinki and was approved by the Ethics Committee of the Saga University Hospital (No. 2023-12-03). Written informed consent was obtained from all patients before their participation.

The operating surgeon for this study, SK, is a highly experienced adult reconstructive surgeon with over 20 years of practice and more than 2000 THA surgeries performed. Starting in May 2023, 34 patients who underwent primary THA were alternately assigned to the control (without VR simulation) or VR (with VR simulation) groups and subsequently operated on by the surgeon (SK).

Methods

To minimize variability in the surgical procedure and acetabular cup placement, the following inclusion and exclusion criteria were applied:

Inclusion Criteria

Patients diagnosed with osteoarthritis or osteonecrosis of the femoral head who were considered eligible for this study underwent primary THA at our institution between May 2023 and October 2023. Only patients who provided informed consent were included.

Exclusion Criteria

Patients were excluded if they had Crowe type III or IV hip dysplasia, required subtrochanteric shortening osteotomy, had ankylosed hips, or had a history of hip surgery or fracture.

Preoperative 3D planning and postoperative assessment using ZedHip^®^

**Figure 1:**
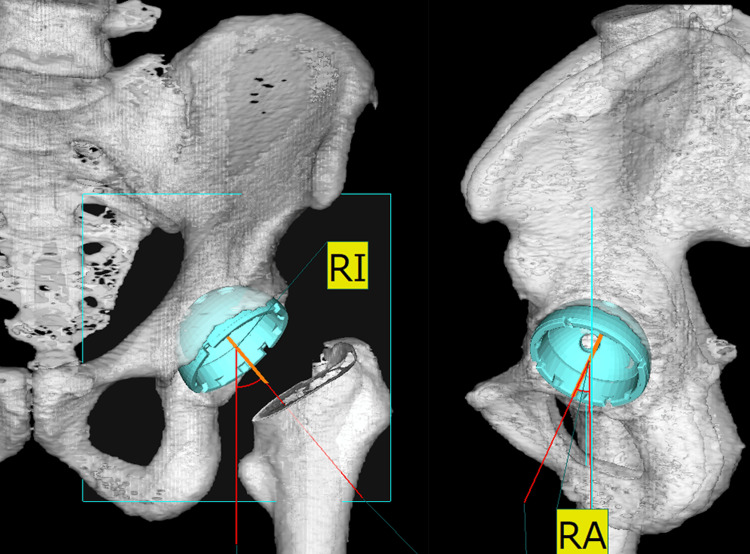
Radiographic visualization of acetabular component orientation The left image depicts the acetabular component orientation, with the RI measured to evaluate the angle of the cup's tilt in the coronal plane. The right image displays the RA, assessing the cup's rotation angle in the axial plane. RA, radiographic anteversion; RI, radiographic inclination

We utilized ZedHip^®^ software (Lexi, Tokyo, Japan) to create three-dimensional (3D) preoperative planning and digital bone models from computed tomography (CT) data. The patients underwent two CT scans preoperatively and one week postoperatively. Using ZedHip^®^ for preoperative 3D planning, we aimed to position the acetabular component to accurately restore the original hip joint center to 40° of inclination (radiographic inclination (RI)) and 20° of anteversion (radiographic anteversion (RA)) relative to the functional pelvic plane (Figure 1).

**Figure 2:**
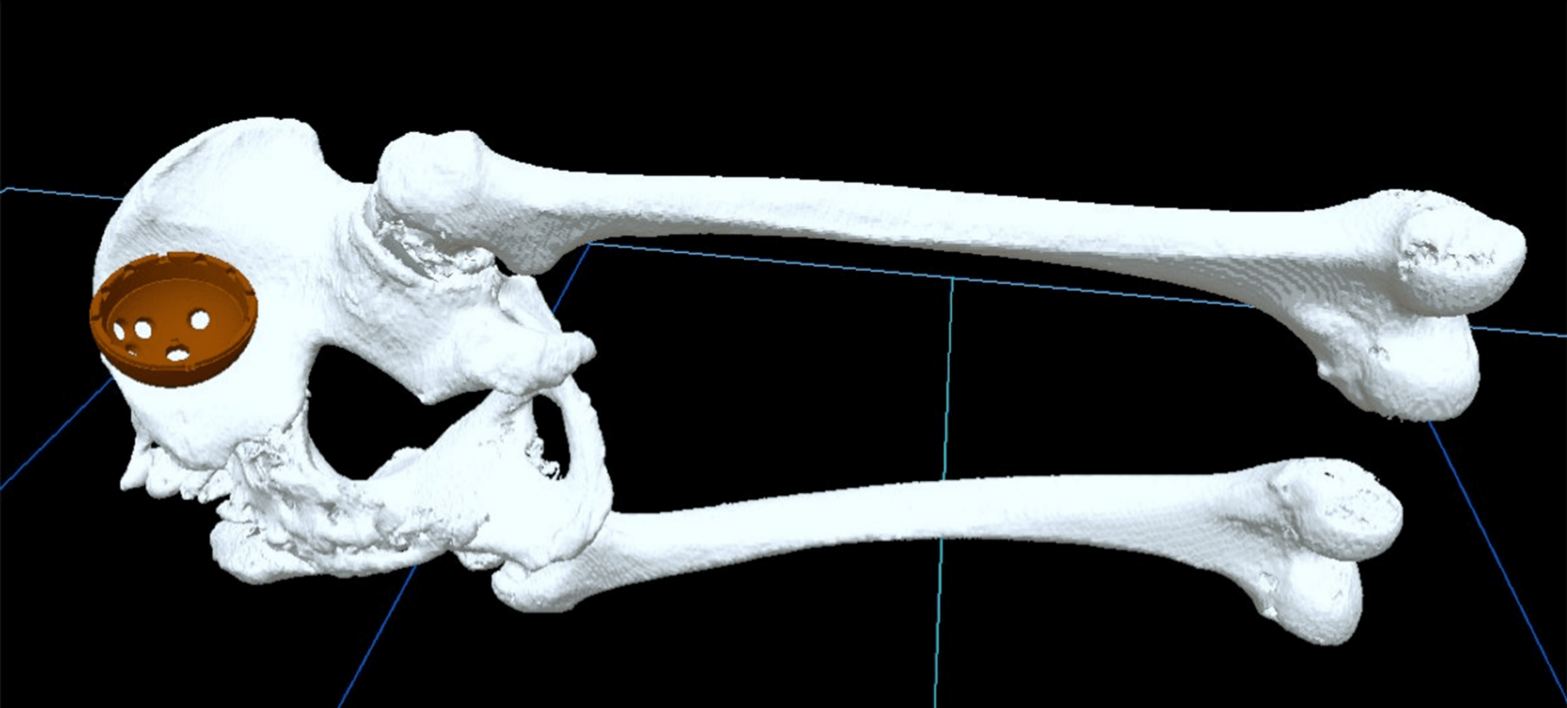
The created VR data: “pelvis,” “femur,” and “cup” VR: virtual reality

The stem was positioned relative to the retrocondylar plane [17], which included the most posterior points of the greater trochanter and the bilateral femoral condyles, and was adjusted to match the shape of the medullary canal of the femoral shaft. Subsequently, stereolithography (STL) files were generated from the ZedHip® planning, producing four sets of VR data: the “pelvis,” “femur,” and “cup,” which were of the same size as used in preoperative planning, and the “pelvis with the cup,” where the cup was positioned according to the preoperative plan (Figure 2).

One week postoperatively, the ZedHip^®^ implant placement assessments were performed based on a second CT scan. This evaluation included a measurement of the RI and RA angles to evaluate deviations (RI and RA differences) from the preoperative plan. Additionally, discrepancies in cup position in the mediolateral, anteroposterior, and superoinferior planes, as well as differences in stem anteversion angle and positioning in the mediolateral and superoinferior planes were measured. Leg length discrepancies and variations in the greater trochanter distances were also assessed.

Implants

For all hip replacements, two types of cementless stems were used: the PerFix Collared Stem (Kyocera Global, Kyoto, Japan), a fit-and-fill type, and the INITIA Stem (Kyocera Global), a taper-wedge type. These were complemented by an SQRUM cup (Kyocera Global), Aquara SQRUM cross-linked ultra-high-molecular-weight polyethylene liner (Kyocera Global), and AZUL ceramic femoral head with a diameter of 32-mm or 28-mm diameters. All the implants were supplied by Kyocera (Osaka, Japan). The choice of stem type was determined preoperatively by the surgeon (SK) based on patient-specific anatomical considerations, age, and body weight.

Virtual reality integration in surgical planning

**Figure 3:**
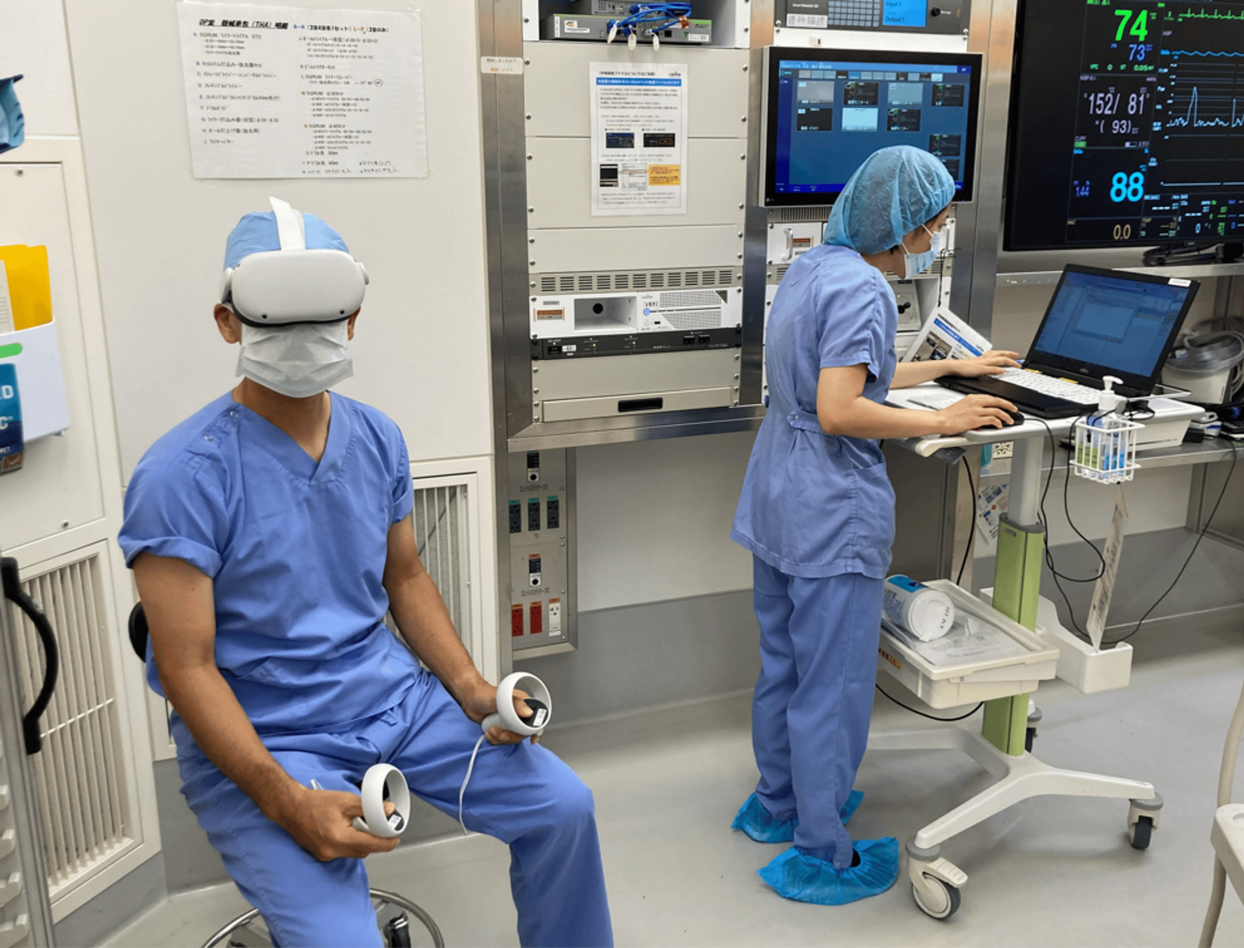
The surgeon in the operating room immediately preoperatively, utilizing HMDs for a final review of the preoperative VR simulation HMD: head-mounted display; VR: virtual reality

**Figure 4:**
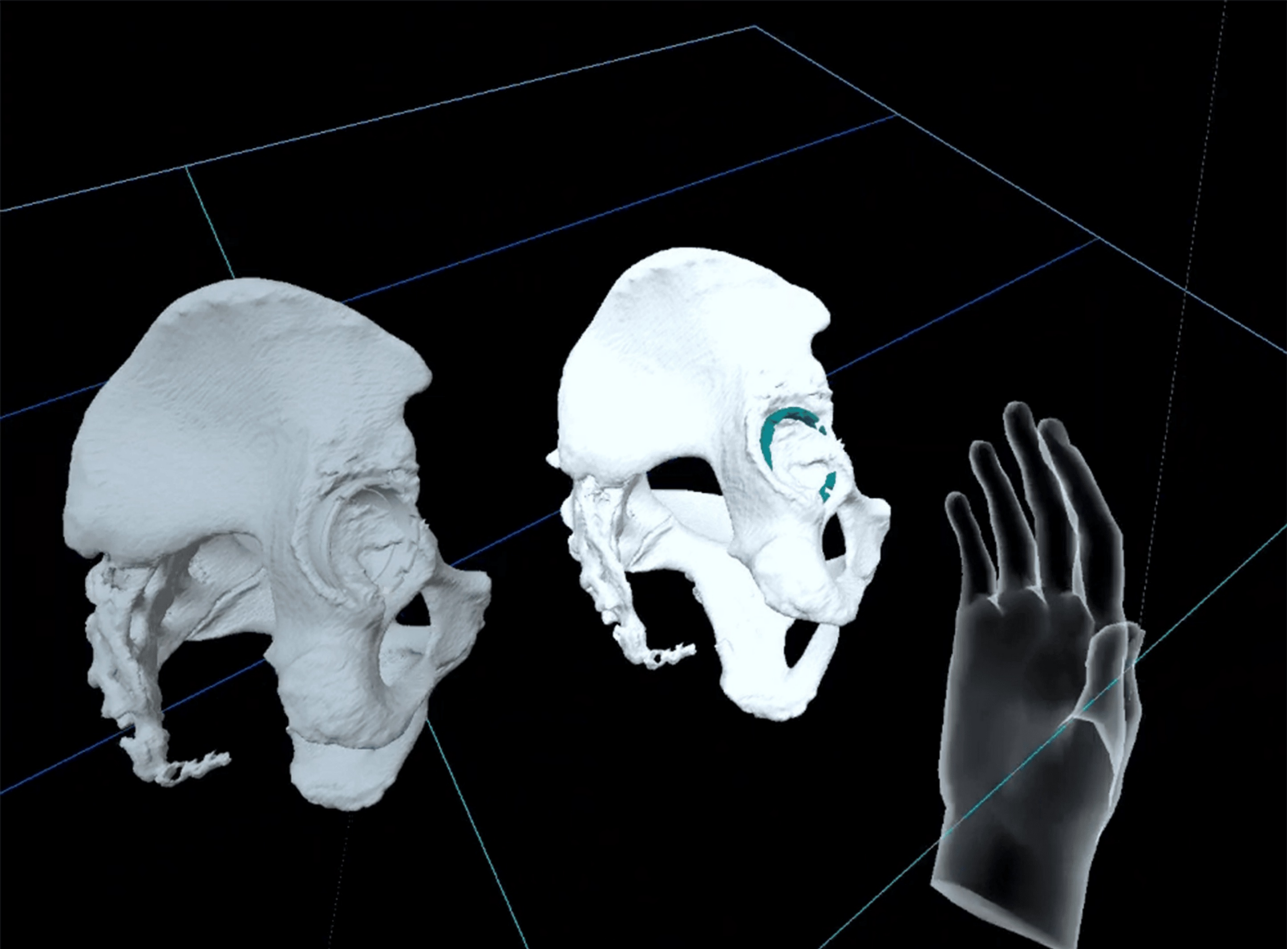
A screenshot from HMDs within the VR environment showcasing the positioning of the “cup” onto the virtual “pelvis,” with the “pelvis with the cup” serving as a reference HMD: head-mounted display; VR: virtual reality

After preoperative 3D planning, the generated 3D STL data were converted to VR data using Holoeyes MD^®^ (Holoeyes Inc, Tokyo, Japan). Subsequently, these data were downloaded onto Meta Quest 2^®^ (Meta Platforms, Inc., Menlo Park, CA, USA). Immediately preoperatively, the surgeon wore the HMDs in the operating room and, using the “pelvis with the cup” as a reference, positioned the “cup” onto the “pelvis” (Figures 3, 4).

**Video 1:** Virtual reality simulation of acetabular cup positioning in THA This video demonstrates the real-time application of VR technology to position the acetabular cup accurately within a preoperative plan. The footage showcases the surgeon’s viewpoint using HMD technology, emphasizing the interactive VR environment and the precision it affords in surgical planning for THA. HMD: head-mounted display; VR: virtual reality; THA: total hip arthroplasty

Additionally, the surgeon reviewed the VR datasets of both the “pelvis” and the “femur” to assess their morphology and ensure optimal placement and alignment. This sequence of actions with the HMDs constituted part of the surgical preparation and was completed within approximately three minutes.

Surgical technique

All THAs were performed by SK via a piriformis-sparing posterior approach under spinal anesthesia using a standardized surgical technique. In all cases, implant placement was performed manually. Notably, intraoperative fluoroscopy or radiographic imaging was not used for confirmation during the procedures. Additionally, navigation or robotic assistance was not employed in any of the cases, ensuring that all placements relied solely on the surgeon's expertise and the mechanical alignment guide. The serial surgical procedures following implant placement consisted of manual joint stability testing, irrigation, capsulotomy site repair, short external rotator muscle repair, superficial bursa of the external rotator muscle repair, fascial repair, and sutures for the subcutaneous and skin layers. Suction drains were used in all the cases. All patients followed the same clinical pathway, including standard post-operative care and antithrombotic therapy.

The primary outcomes of this study were the accuracy of the acetabular cup and femoral stem placement, operative time, intraoperative blood loss, leg length discrepancy, and variation in the distance of the greater trochanter. As secondary outcomes, we further evaluated the postoperative hospital stay and the occurrence of in-hospital complications. Furthermore, the study examined the consistency between the implant sizes specified in the preoperative plans and those used during surgery, focusing on any discrepancies in size for both the acetabular cups and femoral stems.

Statistical analysis

All numerical data are expressed as means ± standard deviation. The two-tailed student’s t-test or Mann-Whitney U test was used to compare two continuous parameters, depending on their distribution and homoscedasticity. Fisher's exact test was used to compare categorical parameters. The significance level was set at p<0.05 for all tests. All analyses were performed using the GraphPad Prism 9 software (GraphPad Software, La Jolla, CA, USA).

## Results

The final analysis included 34 patients (control group, n = 18; VR group, n = 16) for whom the demographic and baseline characteristics are presented in Table 1.

**Table 1:** Demographic and clinical characteristics of the study participants Data are presented as means ± standard deviations for continuous variables and counts for categorical variables. The p-values were calculated using the student's t-test for continuous variables and a two-sided Fisher's exact test for categorical variables. VR: virtual reality

Variable	Control (n=18)	VR (n=16)	p-value
Age	71.8 ± 8.6	66.3 ± 11.4	0.1178
Men/Women	3/15	2/14	1
Body mass index (kg/m^2^)	25.0 ± 3.2	23.5 ± 4.2	0.246
Osteoarthritis/osteonecrosis of the femoral head	17/1	15/1	1
PerFix/INITIA	11/7	9/7	1

There were no significant differences in age, sex distribution, body mass index, underlying cause of THA (osteoarthritis vs. osteonecrosis of the femoral head), or stem model distribution (PerFix vs. INITIA) between the groups.

**Figure 5:**
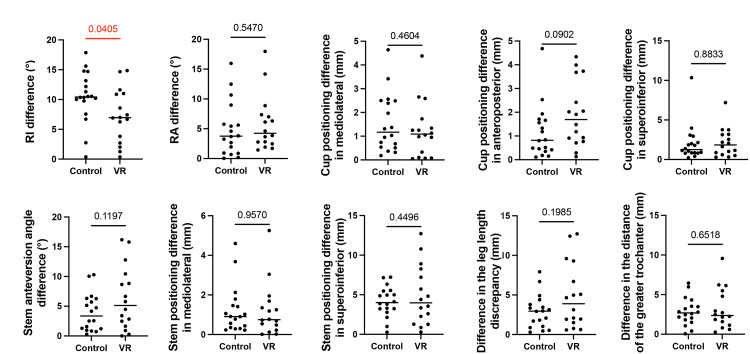
Comparative analysis of radiographic parameters between the Control and VR groups The outcomes of implant positioning parameters between the control and VR groups were analyzed using two-tailed student’s t-tests or Mann–Whitney U tests, depending on the data distribution and homogeneity of variances. Statistical significance was defined as a p-value of <0. VR: virtual reality

The primary surgical outcomes, as assessed using ZedHip^®^, revealed a significant difference in radiographic inclination (RI difference) between the control and VR groups. The control group had a mean RI difference of 10.5 (standard deviation (SD) = 4.3), whereas the VR group showed a mean RI difference of 7.3 (SD=4.5), indicating improved accuracy in the inclination angle with the use of VR (p=0.041). However, no significant differences were observed in radiographic anteversion (RA difference), cup positioning, stem anteversion angle, stem positioning, leg-length discrepancy, or trochanter distance (Figure 5).

The operation time and intraoperative blood loss were also comparable between the two groups (Table 2).

**Table 2:** Comparison of surgical outcomes between the control and VR groups Values are presented as means ± standard deviations, and p-values were calculated using the student's t-test. VR: virtual reality

Surgical Outcome	Control (n=18)	VR (n=16)	p-value
Operation time (min)	46.0 ± 5.1	46.0 ± 4.5	0.9747
Intraoperative blood loss (g)	203.3 ± 77.0	236.1 ± 107.1	0.3091
Postoperative hospital stays (days)	13.39 ± 2.9	13.38 ± 4.0	0.9908

In terms of secondary outcomes, there was no significant difference in the postoperative hospital stay between the groups (Table 2). Neither group exhibited in-hospital complications such as anemia, dislocation, infection, or fracture.

This study further investigated discrepancies between preoperative planning and actual implant sizes used. Discrepancy rates between the planned and actual cup sizes were 33.3% and 25% in the control and VR groups, respectively. Stem size rates were 38.9% and 31.3% in the control and VR groups, respectively (Table 3).

**Table 3:** Discrepancies between planned and actual implant sizes The frequency of discrepancies in implant sizes, comparing the control group to the VR group. For both the cup and the stem, the number of cases where the actual size differed from the planned size is presented, with associated percentages. The p-values were calculated using Fisher's exact test to determine the statistical significance of the observed differences between the two groups. VR: virtual reality

Implant Size Discrepancy	Control (n=18)	VR (n=16)	p-value
Cup size discrepancies	6 (33.3%)	4 (25%)	0.7146
Stem size discrepancies	7 (38.9%)	5 (31.3%)	0.7289

Specifically, in the control group, the acetabular cup was one size smaller in one case and one size larger than planned in five cases. For the femoral stems, one was smaller and six were larger than planned. In the VR group, the cup was one size smaller in one case and one size larger in three cases, whereas the stem was one size larger in four cases. Additionally, in one case, the stem model was changed from INITIA to PerFix.

## Discussion

Overall, the results of this study showed that observation of the patient's 3D bone model in VR immediately and preoperatively notably enhanced the accuracy of acetabular cup placement in terms of RI, demonstrating its effectiveness, even in surgeons with extensive experience. However, this was the only significant difference found, as other aspects of implant positioning, such as surgical smoothness indicators (e.g., operation time and blood loss) and clinical outcomes (e.g., postoperative hospital stay and complications), did not exhibit significant variations between the VR and control groups.

In our study, the VR experience improved the surgeon's comprehension of the patient's specific anatomical structures and provided interactive practice in component placement that could potentially increase surgical precision, even for experienced surgeons. Previous studies have supported these findings, demonstrating that VR with HMDs significantly enhances anatomical comprehension compared with 2D monitors, resulting in better spatial awareness, memory, and model engagement [18]. Our system diverges from previous VR applications that focused primarily on trainee education [12-14,16], as it highlights the development of patient-specific 3D bone models and precise component positioning closely aligned with preoperative planning. As VR technology has become increasingly usable and accessible, its applications have expanded from trainee education to broader clinical use, indicating that VR will be increasingly employed to review complex anatomical data, thus facilitating individualized patient care.

Previous studies have indicated that the RI often deviates more than intended when using the posterior approach and conventional methods [19,20]. Considering this tendency, surgeons in our study aimed to achieve a lower RI. Estimating RI can pose challenges when employing a piriformis-sparing posterior approach, as this technique typically obscures the superior aspect of the acetabulum, owing to the presence of the piriformis muscle. However, the VR experience provides 3D visualization of the anatomy, enabling surgeons to better assess and adjust the acetabular cup position. This ensures closer adherence to the preoperative plan and overcomes visual limitations. Moreover, VR has the potential to provide benefits beyond those of the posterior approach. It can also help overcome the disadvantages associated with other surgical approaches by providing enhanced visualization and planning capabilities that improve overall surgical precision.

One study by Sariali et al. revealed that the 3D templating group achieved notably higher prediction rates for cup and stem sizes in THA, with success rates of 100% and 96%, respectively, compared with only 43% in the conventional 2D group. The 3D group also demonstrated significant accuracy in planning the leg lengths and femoral offset [21]. We hypothesized that utilizing preoperative planning in VR based on 3D templating would improve implant sizing precision. However, our study did not confirm this expectation, potentially because of limitations in the preoperative planning process. In the current setting, preoperative planning was performed on a 2D computer monitor, which does not fully exploit the enhanced spatial understanding gained by viewing the pelvis and femur in an immersive 3D VR environment. If preoperative planning could be conducted directly within a VR environment, more precise and accurate surgical plans could be obtained, potentially improving the accuracy of implant size selection.

Consistent with the findings of Domb et al., our study found that VR did not achieve a higher accuracy in cup placement than robot (RA) or computer-assisted (CA) THA [22]. Interestingly, RA-THA and CA-THA did not show clinically significant differences in length of stay or nonhome discharge rates compared with manual THA but did result in higher in-hospital costs [23]. Both RA-THA and CA-THA require significant equipment and additional personnel, thereby posing resource challenges [15]. Nevertheless, RA and CA THA are expected to become more prevalent in the field of orthopedics. However, our study shows that VR technology offers an equally important but different path of advancement.

The use of patient-specific 3D bone models in VR significantly enhances surgical precision across a broad spectrum of orthopedic procedures. The implications of this technology extend far beyond the boundaries of a single surgical intervention, highlighting its versatility and potential for enhancing a multitude of orthopedic surgeries. This represents a unique trajectory in the advancement of orthopedic surgery, offering a versatile tool with the capability to revolutionize multiple orthopedic procedures.

The absence of significant differences in blood loss or operating time when using VR suggests that the surgeon's expertise combined with established protocols and procedural familiarity may have mitigated the potential time-saving effects of VR. However, for less experienced surgeons, VR could represent a way to reduce operative time, consistent with previous studies [12,13]. Most importantly, no additional patient risk was associated with the use of VR [16], underscoring its value in improving surgical practice. This technology is particularly beneficial for less-experienced surgeons, potentially shortening the steep learning curve associated with surgical procedures.

This study has a primary limitation concerning the simulation process. First, as two different software applications were used, it was not possible to ascertain the angles of anteversion and inclination when positioning the cup in a VR environment. Furthermore, the process only allowed visual assessment of the stem and femur without any simulation of bone osteotomy or stem placement. The future evolution of software is expected to address these limitations, which holds promise for enabling more integrated and comprehensive simulations.

## Conclusions

The use of advanced preoperative VR in surgical preparation demonstrates a significant enhancement in the precision of total hip arthroplasty, particularly concerning the placement of the acetabular cup, even for experienced surgeons. To our knowledge, this is the first study to delineate a promising trajectory for VR technology, not only as a sophisticated training tool but also as a critical component in elevating surgical accuracy in various orthopedic surgeries. These findings suggest that VR technology could be instrumental in advancing both the educational framework and the clinical execution of orthopedic interventions.
